# Levels and Predictors of Suicide Literacy and Suicide Stigma in Spanish‐Speaking Individuals

**DOI:** 10.1002/brb3.70125

**Published:** 2024-11-05

**Authors:** Maitena Pierantonelli, Adriana Mira, Ángel Zamora, Lorena Desdentado, Rebeca Diego‐Pedro, Edgar González‐Hernández, Juana Bretón‐López, Azucena García‐Palacios, Rosa M. Baños

**Affiliations:** ^1^ Polibienestar Research Institute University of Valencia Valencia Spain; ^2^ Department of Personality, Evaluation and Psychological Treatment, Faculty of Psychology University of Valencia Valencia Spain; ^3^ CIBERObn Physiopathology of Obesity and Nutrition Instituto de Salud Carlos III Madrid Spain; ^4^ Faculty of Health Sciences Valencian International University (VIU) Valencia Spain; ^5^ Psychology Department Universidad de las Américas Puebla (UDLAP) Puebla Mexico; ^6^ Department of Basic, Clinical Psychology, and Psychobiology Universitat Jaume I Castelló de la Plana Spain

**Keywords:** Latin America, Spain, Spanish‐speaking individuals, suicide literacy, suicide stigma

## Abstract

**Background:**

One of the obstacles to seeking help for suicide is its social stigma. The literature suggests that suicide knowledge could impact this stigma. The present study aims to examine levels and predictors of suicide stigma and suicide literacy among Spanish‐speaking individuals from Spain and Latin America.

**Method:**

A total of 678 adults completed an online survey conducted from December 2021 to May 2022. The survey assessed sociodemographic and clinical variables, including the Literacy of Suicide Scale (LOSS‐SF) and Stigma of Suicide Scale (SOSS‐SF).

**Results:**

Results showed the presence of stigmatization and a moderate level of suicide literacy. Latin Americans presented significantly more stigmatizing attitudes and lower levels of suicide literacy than Spaniards. Older age and stronger religious/spiritual beliefs were correlated with lower suicide literacy. Suicide stigma was regressed on lower suicide literacy, gender (men), stronger religious/spiritual beliefs, and lacking knowledge about how to find help. Furthermore, the region was significantly associated with the suicide glorification subscale, reporting Latin Americans' greater glorification.

**Conclusions:**

By examining these factors, we aim to foster a deeper understanding of the attitudes and beliefs toward suicide. This understanding is crucial, as it may inform the development of effective interventions and prevention strategies that are culturally sensitive and tailored to diverse populations.

## Graphical abstract

Lower levels of suicide literacy were significantly correlated with higher suicide stigma, particularly among individuals with stronger religious or spiritual beliefs. Factors such as older age, stronger religious or spiritual beliefs, and being male were associated with lower suicide literacy and higher suicide stigma. The research highlights that Latin Americans reported higher levels of suicide glorification, emphasizing the need for culturally sensitive interventions to address suicide stigma and improve suicide literacy.

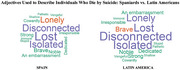

## 1 Introduction

According to the World Health Organization (WHO 2021), suicide is a significant public health issue. Globally, it ranks among the top 20 leading causes of mortality, and it is the second leading cause of death for adolescents and young adults. The latest official report by the Spanish National Statistics Institute (INE 2022) states that the number of deaths by suicide is on the rise every year in Spain. In 2022, suicide remained the leading cause of unnatural death, with an increase of 5.1% over the previous year. Similarly, in Latin America, suicide rates ranged from 2.1 (Venezuela) to 18.8 (Uruguay) per 100,000 people in 2019 (Dattani et al. 2023). One of the major challenges facing suicide research is destigmatization. Addressing this issue is of utmost importance to have the accurate data needed to improve understanding of suicidal behavior and to develop effective preventive interventions (Silvermany 2016).

Suicide prevention is a significant mental health challenge worldwide (Gabilondo 2020). The main barriers to suicide prevention include stigma, lack of information, and considering suicide a taboo subject (WHO 2021). Stigma toward suicide leads to increased symptoms of sadness, distress, and isolation, hindering help‐seeking behavior and impeding suicide prevention (American Psychiatric Association 2021). Public stigma involves negative attitudes, beliefs, and stereotypes held by members of society toward, in this case, people who experience suicidal ideation or behavior (Corrigan et al. 2005). They are often considered selfish, weak‐willed, and unable to cope with problems (Carpiniello and Pinna 2017). In addition, they can elicit emotional responses characterized by anger and, more significantly, fear (Ludwig et al. 2020). These negative perceptions significantly fuel self‐stigma, fostering the internalization of societal misconceptions (Corrigan et al. 2005). Self‐stigma was associated with decreased self‐esteem and self‐efficacy (Corrigan and Bink 2016) and increased symptomatology (Livingston and Boyd 2010). Evidence suggests that individuals with mental health problems who suffer discrimination and perceived stigma may be more prone to suicidal ideation. Studies such as those by Oexle et al. (2017, 2018) have found that self‐stigma mediates in this relationship in the short and long term. Literacy about suicide has been identified as a factor that could not only mitigate the effects of stigma (Peel, Buckby, and McBain 2017; Takahashi et al. 2023) but also increase help‐seeking behaviors (Calear, Batterham, and Christensen 2014; Mok et al. 2021). The concept refers to public knowledge about this problem (i.e., causes, risk factors) (Jorm 2000) and is closely related to educational level, gender, age, previous exposures to close experiences, and cultural context, among others (Batterham, Calear, and Christensen 2013a). Likewise, it was found that peer support networks with higher literacy about suicide tend to encourage professional help‐seeking rather than self‐help (Cruwys et al. 2018).

While culturally diverse countries such as Australia (Chan et al. 2014), China (Han et al. 2017), Japan (Nakamura et al. 2021), Bangladesh (Arafat et al. 2022), Nepal (Gupta et al. 2023), and Germany (Ludwig et al. 2022) have conducted studies to gauge levels of suicide stigma and literacy, such studies are notably scarce in Spanish‐speaking samples (Hernández‐Torres et al. 2021). However, recent validations of key scales to measure these variables are clear indicators of the growing interest in the topic both in Spain (Collado et al. 2023) and Latin America (Hernández‐Torres et al. 2021; Baños‐Chaparro et al. 2023). Understanding attitudes and social thoughts regarding suicide, along with knowledge of the subject, is crucial for developing evidence‐based and culturally sensitive interventions aimed at promoting help‐seeking behaviors when people struggle with suicidality (Calear, Batterham, and Christensen 2014; Batterham et al. 2022).

The aims of the present study were (1) to investigate levels and correlates of suicide stigma and suicide literacy in Spanish‐speaking individuals from Spain and Latin America, comparing both regions; (2) to explore the relationship of sociodemographic and clinical variables with these attitudes and knowledge about suicide by identifying factors that may predict them.

## 2 Materials and Methods

### 2.1 Participants

A total of 678 participants completed an online survey. Sensitivity analyses indicate that the sample size is large enough to detect small effect sizes in the regression models we have run in the study (*R* ^2^ between 0.0268 and 0.0316, specifically). These sensitivity analyses assume a statistical power of 95% and an alpha significance level of 0.05.

### 2.2 Instruments

Ad hoc items were developed to collect sociodemographic information about the sample: gender, age, region, and educational level. The degree of endorsement of religious/spiritual beliefs was determined using a 5‐point Likert scale (*not at all*, *slightly*, *moderately*, *very*, and *extremely*). Likewise, participants were asked about experiences of suicide in their environment and knowledge about helpful resources in case of suicidal risk.

Suicide stigma was assessed with the Stigma of Suicide Scale—Short Form (SOSS‐SF; Batterham, Calear, and Christensen 2013b), which is a 16‐item measure using adjectives that prototypically describe a person who commits suicide. The assessment identifies three factors: stigma, isolation/depression, and glorification/normalization. The scale was validated in Spanish (Collado et al. 2023) and Latino populations (Hernández‐Torres et al. 2021; Baños‐Chaparro et al. 2023) with good psychometric properties. Regarding the reliability of the SOSS‐SF dimensions, it was adequate for stigma (*α* = 0.86; *ω* = 0.84) and isolation/depression (*α* = 0.76; *ω* = 0.81), but was questionable for normalization/glorification (*α *= 0.64; *ω* = 0.68).

The Literacy of Suicide Scale—Short Form (LOSS‐SF; Calear et al. 2021) addresses the levels of knowledge about suicide. It contains 12 items organized into four domains: (1) signs and symptoms, (2) causes or nature of suicide, (3) risk factors for suicidal behavior, and (4) treatment and prevention. The total score can be determined by adding up the correct answers, yielding a range of 0–12. The original version presented adequate psychometric properties as well as validation in Spanish and Latino individuals (Collado et al. 2023; Baños‐Chaparro et al. 2023). In this study, split‐half reliability, corrected by the Spearman‐Brown formula, was *ρ* = 0.61.

An independent back‐translation of the SOSS and LOSS from English to Spanish was conducted. This involved a native Spanish‐speaking translator well‐versed in the subject and a bilingual speaker without prior knowledge in this field. No major differences were found between the two English versions. Team members in Mexico and Spain reviewed items for cultural accuracy. Furthermore, a validation process of the measurements was executed(Zamora et al., 2021).

As mentioned, the presence of clinical symptomatology was also evaluated: (1) Patient Health Questionnaire‐9 (PHQ‐9) reflects the DSM‐IV diagnostic criteria for depression in the last 2 weeks. The Spanish version (Familiar et al. 2015; Diez‐Quevedo et al. 2001) showed adequate sensitivity and specificity; (2) Generalized Anxiety Disorder‐7 (GAD‐7) to assess the manifestation of anxiety in the last 2 weeks. The Spanish version showed excellent internal consistency (García‐Campayo et al. 2010); (3) Suicidal Ideation Attributes Scale (SIDAS) assesses the severity of suicidal ideation in the last month. The measure has shown excellent psychometric properties (van Spijker et al. 2014).

### 2.3 Procedure

Using a non‐random sampling method, participants from both regions were recruited between 2021 and 2022. An announcement of the study was disseminated from different Spanish universities (University of Valencia, University Jaume I, and Zaragoza University) and from the University Of the Americas in Mexico. Flyers and social media were used to invite participants to complete an anonymous online survey. To be included, participants had to be over 18 years old and be fluent in Spanish. This research received approval from the Research Ethics Committee of the Community of Aragon.

### 2.4 Data Analysis

Data management and statistical analyses were performed with SPSS 27 and R 4.2.2. First, descriptive statistics were conducted for sociodemographic and study variables for both the total sample and region‐based subsamples. In addition, we analyzed differences in these variables between region‐based subsamples with *χ* ^2^ tests for categorical variables and independent samples *t*‐test for numeric variables. Moreover, a detailed analysis of the frequency with which participants agreed or strongly agreed with each item of the SOSS‐SF was performed. Similarly, analysis on the number of correct responses in the LOSS‐SF was carried out. Pearson correlation analyses were used to find out the relationships between stigma and other variables of interest with the “apaTables” package (Stanley 2021). Finally, three sets of hierarchical linear regression analyses were computed to investigate the role of suicide literacy dimensions (i.e., risk factors, symptoms, causes, and treatment), sociodemographics (i.e., age, gender‐reference category: man, religion, region‐reference category: Spain, help‐reference category: “yes,” and experience with suicide‐reference category: no experience), and clinical (i.e., depression, anxiety, and suicide ideation) variables in explaining the three dimensions of suicide stigma (i.e., stigma, glorification, and depression).

Moreover, a hierarchical Poisson regression analysis was performed to investigate the role of sociodemographics (i.e., age, gender, level of education‐reference category: basic studies, religion, region, help, and experience with suicide) and clinical (i.e., depression, anxiety, and suicide ideation) variables in explaining the suicide literacy. Poisson regression modeling was chosen because it is the most appropriate method for outcome variables that are counted (Cameron and Trivedi 1998), which is the case for the LOSS total score. All regression analyses were computed using the “stats” package (R Core Team 2022). Quantitative predictors were mean‐centered. The alpha level was set at 0.05 for all statistical analyses computed. Missing data were handled with listwise deletion.

The code behind this analysis has been made publicly available at OSF and can be accessed at https://osf.io/ketrn/.

## 3 Results

### 3.1 Sample Characteristics

Descriptive sample information is provided in Table 1. A total of 78.9% (*n* = 534) identified themselves as female; the mean age was 28.10 years (SD = 10.97; range = 18–77). Regarding the region, 412 were from Spain (60.9%) and 264 from Latin America (39.1%). Most Latin American participants were from Mexico (90.5%) and the rest (9.5%) were from other countries. In total, half of the sample (51.3%) had completed university studies at the highest level of education and the majority (62.2%) were employed. 51.8% were not aware of the resources available to seek help in case of problems related to suicide. Moreover, 56.6% of the participants reported having had an experience related to suicide with someone in their environment. Of these, 42.7% reported that the experience ended with the person's death.

**TABLE 1:** Sociodemographic and clinical characteristics.

	Total	Spain (*N *= 412)	Latin America (*N* = 264)	*χ* ^2^ or *t*
Gender (*n*, %)				
Women	534 (78.9)	326 (79.1)	208 (78.8)	6.37
Men	128 (18.9)	76 (18.4)	51 (19.3)	
Non‐binary	7 (1)	7 (1.7)	0	
Other	8 (1.2)	3 (0.7)	5 (1.9)	
Age (years) (M, SD)	28.10 (10.97)	28.54 (10.43)	27.41 (11.82)	1.24
Education (*n*, %)				
Basic studies	133 (19.6)	126 (30.6)	7 (2.7)	118.63***
Vocational training/high school	196 (29)	69 (16.8)	127 (48.1)	
University studies	347 (51.3)	216 (52.6)	130 (49.2)	
Occupation (*n*, %)				0.02
Employed	331 (62.2)	256 (62.4)	74 (61.2)	
Unemployed	201 (37.8)	154 (37.6)	47 (38.8)	
Religious belief (*M*, SD)	2.29 (1.28)	1.87 (1.10)	2.94 (1.28)	−11.24***
Information on sources of aid (*n*, %)				1.51
Yes	326 (48.2)	207 (50.2)	119 (45.1)	
No	351 (51.8)	205 (49.8)	145 (54.9)	
Experience with suicide in the environment (*n*, %)				
No	294 (44.14)	192 (47.76)	101 (38.70)	6.14*
Yes, only suicide attempt	213 (31.98)	116 (28.86)	96 (36.78)	
Yes, death by suicide	159 (23.87)	94 (23.38)	64 (24.52)	
Relationship closeness (*n*, %)				4.70
Very close	154 (41.4)	84 (40.2)	70 (43.2)	
Close	86 (23.1)	57 (27.3)	29 (17.9)	
Not very close	132 (35.5)	68 (32.5)	63 (38.9)	
Depression—PHQ9 (*M*, SD)	7.13 (5.65)	7.37 (5.67)	6.90 (5.64)	0.78
Anxiety—GAD7 (*M*, SD)	4.87 (4.37)	4.02 (3.87)	6.05 (4.77)	−5.42**
Suicidal ideation—SIDAS (*M*, SD)	11.78 (9.51)	10.58 (8.36)	13.47 (10.71)	−1.83

* *p* < 0.05.

** *p* < 0.01.

*** *p* < 0.001.

No indicators of depressive symptomatology were found in the sample according to the mean of PHQ‐9 (Diez‐Quevedo et al. 2001). Furthermore, the mean score on the 7‐item GAD‐7 (García‐Campayo et al. 2010) indicated a minimal level of anxiety severity. The sample showed low suicidal ideation according to the SIDAS (van Spijker et al. 2014).

Significant differences were found between the Spaniards and Latin Americans in some variables. Latin American participants presented significantly higher levels of anxious symptomatology and religious/spiritual beliefs. Likewise, this group indicated significantly more experiences in the environment related to suicide. Significant differences were also found in terms of the educational level.

### 3.2 Levels of Suicide Stigma and Suicide Literacy

Table 2 shows the means, standard deviations, and the contrast of means by region in both the SOSS‐SF subscales and LOSS‐SF.

**TABLE 2:** Suicide stigma and literacy.

	Total	Spain	Latin America	*t*‐test
Measure	Mean (SD)	Mean (SD)	Mean (SD)	*t*
SOSS‐SF				
Depression/isolation	3.53 (0.83)	3.51 (0.80)	3.57 (0.89)	−0.96
Normalization/glorification	2.69 (0.69)	2.63 (0.70)	2.80 (0.65)	−3.14**
Stigma	1.7 (0.63)	1.65 (0.57)	1.76 (0.69)	−2.13*
LOSS‐SF	6.93 (2.07)	7.12 (1.98)	6.67 (2.18)	2.66**
Causes/nature	2.24 (1.1)	2.39 (1)	2 (1.1)	4.73***
Risk factors	1.4 (0.87)	1.35 (0.82)	1.51 (0.92)	−2.31*
Treatment/prevention	1.91 (0.32)	1.93 (0.29)	1.88 (0.36)	1.69
Signs	1.38 (0.86)	1.45 (0.92)	1.28 (0.9)	2.49*

* *p* < 0.05.

** *p *< 0.01.

*** *p* < 0.001.

Concerning the SOSS‐SF, the results showed that the subscales with the highest scores were depression/isolation and glorification/normalization. In particular, the Latin American group scored significantly higher than the Spaniards on the stigma and glorification/normalization subscales.

**FIGURE 1:**
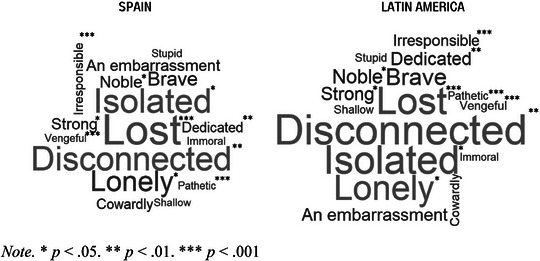
Most agreed to adjectives and significant differences between regions in SOSS‐SF.

Table 3 represents the percentage of agreement with each SOSS‐SF item. The adjectives with the highest agreement were those of the depression/isolation subscale. Specifically, more than half of the participants rated people who commit suicide as “lost,” “disconnected,” and “isolated.” In addition, the Latin American group scored significantly higher on “pathetic.” “vengeful,” “irresponsible,” “isolated,” “disconnected,” “noble,” “strong,” and “dedicated.” Conversely, they showed lower scores in “lost” compared to the Spanish sample (see Figure 1).

**TABLE 3:** Percentage of agreement with SOSS‐SF items.

Item	Agree/strongly agree, %
Lost	68.14%
Disconnected	59.73%
Isolated	57.82%
Lonely	46.61%
Brave	23.45%
Noble	15.93%
Strong	14.16%
An embarrassment	13.27%
Dedicated	11.65%
Cowardly	7.67%
Irresponsible	7.37%
Vengeful	2.36%
Shallow	1.62%
Pathetic	1.33%
Stupid	1.33%
Immoral	1.18%

Regarding the LOSS‐SF, Table 2 shows that the scores in the causes/nature subscale were markedly high, while in the rest of the subscales, the scores were close to half of the maximum score. For the Spaniard group, significantly higher scores were found on the total scale and the subscales of causes/nature and symptoms, while Latin American participants scored significantly higher on the risk factors subscale. Table 4 shows the specific percentage of correct answers for each statement.

**TABLE 4:** Correct responses to items from the LOSS‐SF.

Item	Total (*N* = 678)	Spain (*N* = 412)	Latin America (*N* = 262)
	%	%	%
Causes/nature			
Very few people have thoughts about suicide (F)	80.9%	83.3%	75.6%
If assessed by a psychiatrist, everyone who suicides would be diagnosed as depressed (F)	18.5%	17.1%	19.1%
A suicidal person will always be suicidal and entertain thoughts of suicide (F)	73.8%	78.2%	65.6%
Talking about suicide always increases the risk of suicide (F)	50.4%	56.8%	40.1%
Risk factors			
Most people who suicide are psychotic (F)	67.1%	71.6%	59.2%
Men are more likely to suicide than women (T)	37%	29.6%	47.7%
There is a strong relationship between alcoholism and suicide (T)	36.4%	31.3%	43.9%
Signs			
Not all people who attempt suicide plan their attempt in advance (T)	66.9%	68.9%	61.8%
People who talk about suicide rarely kill themselves (F)	49.8%	55.8%	39.3%
People who want to attempt suicide can change their mind quickly (T)	21.6%	18%	26.7%
Treatment/prevention			
People who have thoughts about suicide should not tell others about it (F)	95.1%	95.1%	93.1%
Seeing a psychiatrist or psychologist can help prevent someone from suicide (T)	95.7%	94.7%	95%

Abbreviations: F, false; T, true.

### 3.3 Relationship Between Sociodemographic and Clinical Variables With Suicide Stigma and Suicide Literacy

Pearson's correlation coefficients between sociodemographics, clinical variables, suicide stigma, and literacy are presented in Table 5.

**TABLE 5:** Bivariate correlations between the suicide stigma, suicide literacy, and clinical variables.

Variable	1	2	3	4	5	6	7	8	9	10	11	12
1. Age												
2. Religious/spiritual beliefs	0.08*											
3. SOSS‐estigma	0.06	0.16**										
4. SOSS‐glorification	−0.06	0.03	0.07									
5. SOSS‐depression	−0.01	0.03	0.31**	−0.02								
6. LOSS‐risk factor	−0.18**	−0.03	−0.10**	0.00	0.07							
7. LOSS‐symptoms	−0.12**	−0.09*	−0.08*	−0.02	0.03	0.18**						
8. LOSS‐causes	−0.26**	−0.17**	−0.23**	−0.01	0.00	0.25**	0.28**					
9. LOSS‐treatment	−0.11**	−0.13**	−0.21**	−0.06	0.03	0.12**	0.17**	0.24**				
10. LOSS‐total score	−0.27**	−0.16**	−0.23**	−0.02	0.05	0.64**	0.66**	0.77**	0.39**			
11. PHQ‐9	−0.09*	−0.15**	−0.08*	0.09*	0.05	0.09*	0.16**	0.03	−0.01	0.12**		
12. GAD‐7	−0.14**	0.04	0.02	0.11**	0.12**	0.05	0.10*	0.02	−0.09*	0.06	0.60**	
13. SIDAS	−0.13	−0.07	0.17*	0.10	0.18*	0.17*	0.14	−0.11	−0.21**	0.05	0.52**	0.35**

* *p *< 0.05.

** *p *< 0.01.

Age correlated significantly and negatively with the total and all subscales of the LOSS scale. In turn, religious/spiritual beliefs exhibited a significant positive correlation with the stigma subscale.

Conversely, these beliefs negatively correlated with the overall total of the LOSS scale and its subscales, except for the risk factors subscale. The stigma subscale showed consistent negative correlations with all aspects of suicide literacy, emphasizing the link between stigma and decreased knowledge of suicide.

The correlation analysis revealed significant relationships between the clinical variables and stigma and their associations with literacy subscales.

Depressive symptoms were positively correlated with the glorification subscale but negatively correlated with overall suicide stigma, indicating a nuanced relationship. Anxiety symptoms were positively associated with various stigma aspects, including glorification, depression, and overall stigma. Suicidal ideation also showed positive correlations with the stigma subscale, depression subscale, and stigma overall, suggesting that higher levels of suicidal ideation were associated with greater endorsement of suicide stigma.

Finally, all clinical variables positively correlated with literacy overall and several subscales.

### 3.4 Stigma of Suicide Scale Dimensions Regressed on Suicide Literacy, Sociodemographic, and Clinical Variables

Regarding the hierarchical linear regression models to explain the SOSS stigma subscale (see Table 6), the model that included the suicide literacy dimensions as explanatory variables was significant (Model 1: *F*(4,533) = 9.03, *p *< 0.001), explaining 5.87% of the variance. Specifically, lower scores on the causes and treatment dimensions of the LOSS scale were significantly related to higher scores on suicide stigma. After simultaneously adding the sociodemographic variables, the resulting model (Model 2: *F*(11,526) = 6.46, *p* < 0.001) produced a significant increase in the explained variance (*∆R* ^2^ = 0.056, *F*(7) = 4.75, *p *< 0.001). In addition to the causes and treatment dimensions of the LOSS scale, gender, religion, and help were also significant predictors of suicide stigma. Specifically, women showed lower levels of suicide stigma than men, whereas those with stronger religious beliefs and those who reported a lack of knowledge about how to find help showed higher levels of suicide stigma. When simultaneously adding the clinical variables (i.e., anxiety, depression, and suicide ideation), the resulting model (Model 3: *F*(14,523) = 5.44, *p *< 0.001) did not produce a significant increase in the explained variance of stigma compared to Model 2 (*∆R* ^2^ = 0.008, *F*(3) = 1.61, *p *< 0.180). The only new significant predictor was depression, demonstrating greater depressive symptoms related to lower scores on suicide stigma.

**TABLE 6:** Linear regression results using SOSS Stigma subscale as the criterion variable.

Model	Predictors	*b* (SE)	*β*	*t*	Fit (*R* ^2^, 95% CI)	*R* ^2^_adjusted_
**1.1**	**(Intercept)**	**1.70 (0.03)**	**—**	**67.27** **	**0.063** ** **[0.02, 0.10]**	**0.056**
	LOSS‐risk factors	−0.04 (0.03)	−0.06	−1.30		
	LOSS‐symptoms	−0.01 (0.03)	−0.01	−0.25		
	LOSS‐causes	−0.10 (0.03)	−0.18	−3.91**		
	LOSS‐treatment	−0.23 (0.09)	−0.11	−2.63**		
**1.2**	**(Intercept)**	**1.84 (0.07)**	**−0.04**	**26.00** ***	**0.119** ** **[0.06, 0.15]**	**0.101**
	LOSS‐risk factors	−0.03 (0.03)	−0.01	−0.95		
	LOSS‐symptoms	−0.01 (0.03)	−0.18	−0.28		
	LOSS‐causes	−0.1 (0.03)	−0.11	−3.84***		
	LOSS‐treatment	−0.23 (0.09)	−0.00	−2.60**		
	Age	0.00 (0.00)	−0.16	−0.01		
	Gender (women)	−0.25 (0.06)	0.14	−3.88***		
	Religion/spiritual beliefs	0.07 (0.02)	−0.05	3.11**		
	Region (Latin America)	−0.06 (0.06)	0.13	−1.11		
	Help (no)	0.16 (0.05)	0.01	3.25**		
	Exp. suicide (attempt)	0.01 (0.06)	0.01	0.15		
	Exp. suicide (death)	0.01 (0.07)	−0.04	0.16		
**1.3**	**(Intercept)**	**1.85 (0.07)**	**—**	**25.60** ***	**0.127** ** **[0.06, 0.16]**	**0.104**
	LOSS‐risk factors	−0.03 (0.03)	−0.04	−0.92		
	LOSS‐symptoms	0.00 (0.03)	−0.01	−0.14		
	LOSS‐causes	−0.1 (0.03)	−0.19	−4.01***		
	LOSS‐treatment	−0.22 (0.09)	−0.10	−2.43*		
	Age	0.00 (0.00)	0.00	0.09		
	Gender (women)	−0.25 (0.06)	−0.16	−3.94***		
	Religion/spiritual beliefs	0.06 (0.02)	0.13	2.69**		
	Region (Latin America)	−0.09 (0.06)	−0.07	−1.49		
	Help (no)	0.16 (0.05)	0.13	3.13**		
	Exp. suicide (attempt)	0.01 (0.06)	0.01	0.23		
	Exp. suicide (death)	0.02 (0.07)	0.02	0.33		
	PHQ‐9	−0.01 (0.01)	−0.13	−2.15*		
	GAD‐7	0.01 (0.01)	0.08	1.52		
	SIDAS	0.00 (0.00)	0.04	0.77		

Abbreviations: *β*, standardized regression weights; *b*, unstandardized regression weights; SE, standard error.

* *p* < 0.05.

** *p* < 0.01.

*** *p* < 0.001.

Concerning the hierarchical linear regression models to explain the suicide glorification subscale (see Table 7), the model that included the suicide literacy dimensions as explanatory variables was not significant (Model 1: *F*(4,533) = 1.43, *p* = 0.223), explaining 1.01% of the variance. The only significant predictor was the treatment dimension of the LOSS scale, with lower scores related to higher scores on glorification. After simultaneously adding the sociodemographic variables, the resulting model was marginally significant (Model 2: *F*(11,526) = 1.66, *p* = 0.078) and produced a marginal increase in the explained variance (*∆R* ^2^ = 0.030, *F*(7) = 5.47, *p* = 0.087). In addition to the treatment dimension of the LOSS scale, the region was also a significant predictor of suicide glorification; thus, Latin Americans reported greater glorification than Spaniards. Finally, when simultaneously adding the clinical variables, the resulting model was still marginally significant (Model 3: *F*(14,523) = 1.57, *p* = 0.085) and did not contribute significantly to explaining the variance of glorification compared to Model 2 (*∆R* ^2^ = 0.006, *F*(3) = 1.56, *p* = 0.311). None of the clinical variables were statistically significant.

**TABLE 7:** Linear regression results using SOSS Glorification subscale as the criterion.

Model	Predictors	*b* (SE)	*β*	*t*	Fit (*R* ^2^, 95% CI)	*R* ^2^_adjusted_
**2.1**	**(Intercept)**	**2.7 (0.03)**	**—**	**94.25** ***	**0.004 [0.00, 0.01]**	**0.003**
	LOSS‐risk factors	−0.03 (0.03)	−0.03	−0.76		
	LOSS‐symptoms	0.03 (0.03)	0.04	0.98		
	LOSS‐causes	0.00 (0.03)	0.01	0.15		
	LOSS‐treatment	−0.21 (0.10)	−0.09	−2.09*		
**2.2**	**(Intercept)**	**2.67 (0.08)**	**—**	**32.76** ***	**0.034 [0.00, 0.05]**	**0.013**
	LOSS‐risk factors	−0.05 (0.03)	−0.06	−1.34		
	LOSS‐symptoms	0.03 (0.04)	0.04	0.90		
	LOSS‐causes	0.01 (0.03)	0.01	0.23		
	LOSS‐treatment	−0.21 (0.10)	−0.09	−2.04*		
	Age	0.00 (0.00)	−0.07	−1.62		
	Gender (women)	−0.03 (0.07)	−0.02	−0.44		
	Religion/spiritual beliefs	−0.03 (0.02)	−0.06	−1.17		
	Region (Latin America)	0.16 (0.07)	0.12	2.40*		
	Help (no)	−0.05 (0.06)	−0.04	−0.94		
	Exp. suicide (attempt)	0.07 (0.07)	0.05	1.10		
	Exp. suicide (death)	−0.01 (0.08)	−0.01	−0.16		
**2.3**	**(Intercept)**	**2.70 (0.08)**	**—**	**32.31** ***	**0.040 [0.00, 0.05]**	**0.015**
	LOSS‐risk factors	−0.05 (0.03)	−0.06	−1.36		
	LOSS‐symptoms	0.02 (0.04)	0.03	0.64		
	LOSS‐causes	0.01 (0.03)	0.01	0.24		
	LOSS‐treatment	−0.19 (0.10)	−0.08	−1.82		
	Age	0.00 (0.00)	−0.07	−1.49		
	Gender (women)	−0.04 (0.07)	−0.02	−0.51		
	Religion/spiritual beliefs	−0.02 (0.02)	−0.05	−0.93		
	Region (Latin America)	0.14 (0.07)	0.10	2.05*		
	Help (no)	−0.06 (0.06)	−0.04	−0.99		
	Exp. suicide (attempt)	0.05 (0.07)	0.03	0.71		
	Exp. suicide (death)	−0.03 (0.08)	−0.02	−0.46		
	PHQ‐9	0.01 (0.01)	0.04	0.64		
	GAD‐7	0.01 (0.01)	0.05	0.88		
	SIDAS	0.00 (0.01)	0.01	0.09		

Abbreviations: *β*, standardized regression weights; *b*, unstandardized regression weights; SE, standard error.

* *p * < 0.05.

** *p* < 0.01.

*** *p * < 0.001.

Regarding the hierarchical linear regression models to explain the depression subscale (see Table 8), the model that included the suicide literacy dimensions as explanatory variables was not significant (Model 1: *F*(4,533) = 1.59, *p* = 0.176), explaining 1.18% of the variance. None of the suicide literacy dimensions reached statistical significance. After simultaneously adding the sociodemographic variables, the resulting model (Model 2: *F*(11,526) = 0.83, *p* = 0.609) did not produce a significant increase in the explained variance *(∆R* ^2^ = 0.005, *F*(7) = 0.041, *p* = 0.898), and none of the predictors reached statistical significance. When simultaneously adding the clinical variables, the resulting model was not significant (Model 3: *F*(14,523) = 1.21, *p* = 0.261), but we found a marginally significant increase in the explained variance of stigma compared to Model 2 (*∆R* ^2^ = 0.014, *F*(3) = 5.17, *p* = 0.052). Anxious symptomatology was the only significant predictor, with higher scores on GAD‐7 related to higher scores on the SOSS depression subscale.

**TABLE 8:** Linear regression results using SOSS Depression subscale as the criterion variable.

Model	Predictors	*b* (SE)	*β*	*t*	Fit (*R* ^2^, 95% CI)	*R* ^2^_adjusted_
**3.1**	**(Intercept)**	**3.54 (0.04)**	**—**	**100.46** ***	**0.012 [0.00, 0.03]**	**0.004**
	LOSS‐risk factors	0.06 (0.04)	0.07	1.50		
	LOSS‐symptoms	0.02 (0.04)	0.02	0.39		
	LOSS‐causes	−0.06 (0.04)	−0.08	−1.73		
	LOSS‐treatment	0.18 (0.12)	0.06	1.45		
**3.2**	**(Intercept)**	**3.57 (0.10)**	**—**	**35.40** ***	**0.017 [0.00, 0.02]**	**0.000**
	LOSS‐risk factors	0.07 (0.04)	0.07	1.61		
	LOSS‐symptoms	0.01 (0.04)	0.01	0.30		
	LOSS‐causes	−0.06 (0.04)	−0.08	−1.67		
	LOSS‐treatment	0.18 (0.13)	0.06	1.42		
	Age	0.00 (0.00)	0.00	0.09		
	Gender (women)	−0.09 (0.09)	−0.04	−0.98		
	Religion/spiritual beliefs	0.02 (0.03)	0.03	0.64		
	Region (Latin America)	−0.03 (0.08)	−0.02	−0.40		
	Help (no)	0.07 (0.07)	0.04	0.99		
	Exp. suicide (attempt)	0.05 (0.08)	0.03	0.65		
	Exp. suicide (death)	−0.01 (0.09)	−0.01	−0.13		
**3.3**	**(Intercept)**	**3.64 (0.10)**	**—**	**35.30** ***	**0.031 [0.00, 0.04]**	**0.005**
	LOSS‐risk factors	0.07 (0.04)	0.08	1.66		
	LOSS‐symptoms	0.01 (0.04)	0.01	0.11		
	LOSS‐causes	−0.07 (0.04)	−0.09	−1.86		
	LOSS‐treatment	0.22 (0.13)	0.08	1.72		
	Age	0.00 (0.00)	0.02	0.37		
	Gender (women)	−0.10 (0.09)	−0.05	−1.13		
	Religion/spiritual beliefs	0.02 (0.03)	0.03	0.57		
	Region (Latin America)	−0.09 (0.08)	−0.06	−1.09		
	Help (no)	0.05 (0.07)	0.03	0.75		
	Exp. suicide (attempt)	0.02 (0.08)	0.01	0.26		
	Exp. suicide (death)	−0.03 (0.09)	−0.02	−0.36		
	PHQ‐9	−0.01 (0.01)	−0.06	−0.95		
	GAD‐7	0.03 (0.01)	0.15	2.66**		
	SIDAS	0.00 (0.010)	0.01	0.23		

Abbreviations: *β*, standardized regression weights; *b*, unstandardized regression weights; SE, standard error.

* *p* < 0.05.

** *p* < 0.01.

*** *p *< 0.001

No severe violations of assumptions of linearity, normality of residuals, and homogeneity of residuals variance were detected in residual versus fitted plots, Q–Q plots, or scale‐location plots, respectively. In addition, variance inflation factors did not indicate concerns about multicollinearity, as the highest value found was 2.34.

### 3.5 Suicide Literacy Regressed on Sociodemographic and Clinical Variables

Regarding the hierarchical Poisson models to explain suicide literacy (total score) (see Table 9), the model that included sociodemographic characteristics as explanatory variables was significant (Model 4.1: *χ* ^2^(9) = 29.26, *p* = 0.001), explaining 8.44% of the variance. Religion/spiritual beliefs were the only significant predictor in this model, with stronger beliefs being associated with lower suicide literacy. After simultaneously adding the clinical variables, the resulting model (Model 2: *χ* ^2^(12) = 30.54, *p* = 0.002) did not produce a significant increase in the explained variance (*∆R* ^2^ = 0.004, *∆χ* ^2^ = 1.28, *p* = 0.733). None of the clinical predictors were statistically significant. The Q–Q plots did not suggest the violation of the normality of residuals.

**TABLE 9:** Poisson regression results using LOSS Total score as the criterion variable.

Model	Predictors	*b* (SE)	*β*	*z*	Fit (*R* ^2^)
**4.1**		1.96 (0.06)	—	34.97***	0.084
	Age	−0.01 (0)	−0.04	−4.23***	
	Gender (women)	−0.05 (0.04)	−0.01	−1.12	
	Education (vocational training)	0 (0.05)	0.00	−0.04	
	Education (university)	0.05 (0.05)	0.01	1.18	
	Religion/spiritual beliefs	−0.03 (0.01)	−0.02	−1.97*	
	Region (Latin America)	−0.04 (0.04)	−0.01	−1.03	
	Help (No)	−0.01 (0.03)	0.00	−0.45	
	Exp. suicide (attempt)	0.04 (0.04)	0.01	0.98	
	Exp. suicide (death)	0.02 (0.04)	0.00	0.51	
**4.2**	(Intercept)	1.97 (0.06)	—	34.68***	0.088
	Age	−0.01 (0)	−0.04	−4.12***	
	Gender (women)	−0.05 (0.04)	−0.01	−1.17	
	Education (vocational training)	0 (0.05)	0.00	−0.06	
	Education (university)	0.06 (0.05)	0.01	1.21	
	Religion/spiritual beliefs	−0.03 (0.01)	−0.02	−1.87	
	Region (Latin America)	−0.05 (0.04)	−0.01	−1.12	
	Help (No)	−0.02 (0.03)	0.00	−0.55	
	Exp. suicide (attempt)	0.03 (0.04)	0.01	0.78	
	Exp. suicide (death)	0.01 (0.04)	0.00	0.35	
	PHQ‐9	0 (0)	0.00	0.34	
	GAD‐7	0 (0)	0.01	0.75	
	SIDAS	0 (0)	−0.01	−0.49	

Abbreviations: *β*, standardized regression weights; *b*, unstandardized regression weights; SE, standard error.

* *p* < 0.05.

** *p* < 0.01.

*** *p *< 0.001

## 4 Discussion

The present study aimed to examine the levels and correlates of suicide stigma and suicide literacy in Spanish‐speaking people, an understudied phenomenon in this population. First, results showed the presence of stigmatization, a fact that is in line with previous research collected by Carpiniello and Pina (2017), which demonstrated the presence of these negative attitudes in a diversity of sociocultural environments. Mainly, a predominance of the conception of suicide as a typical act of people with traits related to isolation and depression was observed; the most prevalent adjectives were “lost,” “disconnected,” and “isolated.” This is not surprising because feelings of isolation and loneliness are known risk factors for suicide, whereas feelings of connectedness with family and community are known to be protective (Centers for Disease Control and Prevention Suicide Prevention 2022). These results are in line with the work of Batterham et al. (2019) in Australia, Han et al. (2017) in China, Al‐Shannaq and Aldalaykeh (2021) in Jordan, and Nakamura et al. (2021) in Japan.

Regarding regions, the Latin American (vs. the Spaniard) group showed more stigmatizing attitudes and scored significantly higher on the stigma and glorification/normalization subscales. Notably, participants from this group associated individuals who die by suicide with descriptors like “noble” and “strong.” This normalization of suicide has been linked with a decline in help‐seeking behaviors and an elevation in suicidality (Oexle et al. 2022). In suicide literacy programs, it is important to demystify the act of suicide, reduce its glorification, and facilitate coping strategies and alternative options.

In addition, it is an interesting result since differences in culture and religion could explain these positive connotations of suicide. Notably, most of the participants from Latin America came from Puebla in Mexico, who share a spiritual understanding of death accompanied by rituals and a strong religious tendency toward Catholicism(Gutierrez et al., 2020). This would also explain the difference in religious beliefs between the samples. Likewise, this study revealed that participants from Latin America exhibited significantly more anxiety symptoms and experiences related to suicide in their environment. These findings suggest a possible connection to the mental health access gap in the region, resulting in a lack of adequate care for those in need (Kohn et al. 2018).

The overall score of participants on the LOSS‐SF suggested a moderate level of suicide literacy. Specifically, the scores in the causes/nature subscale were markedly high, while in the rest of the subscales, the scores were close to half of the maximum. Likewise, the results found in this sample are similar to the means reported in Japan (Nakamura et al. 2021) or Germany (Ludwig et al. 2022). On the contrary, they indicate lower levels of literacy compared to the Australian sample (Batterham, Calear, and Christensen 2013a), but better results than those replicated in China (Han et al. 2017).

Overall, the Latin American population presented significantly lower levels of suicide literacy. Therefore, the observed differences between regions in terms of suicide knowledge may be explained by differences in the education about mental health, specifically in the causes of suicide, its treatment, and the risk factors. This might help to design the specific components that interventions against stigma must have depending on where they will be implemented.

Furthermore, the present study identified associations between age and literacy level. The lowest levels of knowledge were found among those with higher ages. It could be explained by the greater access to information that younger people have. Moreover, our findings showed that stronger religious/spiritual beliefs are associated with lower suicide literacy. This finding is aligned with previous studies indicating variations in mental health literacy according to religious beliefs, highlighting this sociodemographic variable as an important factor to be considered (Furnham and Swami 2018). Therefore, according to these results, the intensity of religious beliefs could not only increase the stigma toward suicide but also reduce knowledge about the subject.

Another important finding was that suicide literacy was associated with suicide stigma. Individuals with a higher degree of knowledge were less prone to stigmatizing suicide, a finding supported by previous studies in Australia (Batterham, Calear, and Christensen 2013a), Germany (Ludwig et al. 2022), and Japan (Nakamura et al. 2021). Particularly, greater knowledge about the causes and treatments of suicide was associated with a lower presence of stigmatizing attitudes. These results reinforce what has been stated in recent meta‐analyses and reviews on the subject, justifying the usefulness of implementing universal prevention programs based on suicide literacy (Hofstra et al. 2020) for reducing stigma and increasing help‐seeking attitudes (Calear, Batterham, and Christensen 2014), contributing to the main purpose: the prevention of suicide.

As regards the relationship between sociodemographic, clinical, and literacy variables with the stigma subscale, our results from the regression models were very similar to the study from Germany, where Ludwig et al. (2022) explained 13% of the variance. As observed in the literature, both in the general population (Batterham, Calear, and Christensen 2013a; Batterham et al. 2019; Pereira and Cardoso 2019) and in the clinical population (Batterham et al. 2019), men had higher levels of stigmatizing attitudes than women. This relationship is justified in the literature based on the possible presence of a lower level of knowledge about suicide in males (Batterham, Calear, and Christensen 2013a). Alternatively, the possible influence of traditional masculine ideals, such as stoicism, self‐sufficiency, or restricted emotionality, could lead to a lower predisposition to seek help and increase the presence of self‐stigma and, consequently, public stigma (Mackenzie et al. 2019). According to our results, lack of knowledge about sources of help in cases of suicidal ideation was associated with higher levels of stigma. This association could partially explain the inhibition of help‐seeking behaviors for mental health problems (Clement et al. 2015). The reduction of stigma could increase help‐seeking behavior. As pointed out in a recent systematic review of the factors associated with professional help‐seeking for suicidality (Han et al. 2018), stigmatizing attitudes toward suicide and low suicide literacy are important barriers to professional help‐seeking. In our sample, it was observed that more than half of the participants did not know where to ask for help in both regions. This is an alarming fact since the prevalence of suicidal ideation in the current study was 22.7%, and a large number of suicides could be prevented if people experiencing suicidal thoughts or behaviors sought support from appropriate health services (Hom, Stanley, and Joiner 2015). It should be noted that at the end of the evaluation survey, they had several telephone contacts to ask for help.

Our results summarize that, in Spanish‐speaking individuals, the three dimensions of suicide stigma could be explained by one or several study variables. Specifically, higher levels of suicide stigma were positively related to lack of knowledge about the topic (i.e., the causes and treatment or prevention), male gender, and adherence to religious beliefs. In addition, it was found that those who were unaware of sources of help for suicidal thoughts or behaviors also showed greater stigma toward suicide.

Regarding suicide glorification, it should be noted that it varied across regions, with Latin Americans showing higher scores than Spanish people.

Finally, as regards depression/isolation‐related suicide stigma, it tended to be greater in those who suffered greater anxious symptomatology.

The positive relationships found between clinical variables and the stigmatization of suicide may indicate a possible internalized stigma (Batterham, Calear, and Christensen 2013a). This relationship could arise due to the interpretation of the experiences of others based on our own experiences. Specifically, the higher presence of suicidal ideation corresponded to a higher tendency toward stigmatization, in line with previous literature (Batterham et al. 2019), a result that highlights the possibility that this type of attribution could be indicative of a higher risk of suicide (Batterham et al. 2019), where the combination of low knowledge and high glorification of suicide could make it difficult for people with ideation to seek help.

It is important to note that these results should be interpreted with caution. Adding the clinical variables did not produce a significant increase in the explained variance of stigma, being 10% in this study. Thus, the results also suggested that other important factors should be taken into account when addressing the stigma of suicide. For example, variables such as suffering from a mental disorder, a close person who died by suicide, or some cultural values related to suicide stigma (Mascayano et al. 2015).

Our results suggest the need to work against stigma by promoting knowledge of the phenomenon, aiding resources, and suicide awareness. They are in line with the literature that points out the important role of knowledge in the production of the stigmatization process (Gronholm et al. 2017). However, some limitations of the current study should be mentioned. Due to the sampling method used, the sample may not be completely representative of the Spaniard and Latin American populations. In addition, there are differences between the two samples in several aspects, such as educational level, although this also reflects differences at the population level between Spain and Mexico (Organisation for Economic Cooperation and Development 2022). On the other hand, there was an overrepresentation of women and individuals in the emerging adulthood stage. Given the stigma surrounding the research topic, self‐selection bias may have impacted the sample (although similar limitations applied to the Australian and Japanese samples). The cross‐sectional design of the study precludes establishing causality in the relationships examined. The sample exhibited low levels of clinical symptoms, which hinders drawing firm conclusions about the association between mental health problems and suicide stigma.

Future studies would benefit from including heterogeneous sociodemographic profiles that carry greater variability and accurately demonstrate suicide stigma, literacy, and its potential determinants.

## 5 Conclusions

Our findings suggest that suicide literacy and some sociodemographic variables (e.g., gender, region) are associated with suicide stigma among a Spanish‐speaking population. This study constitutes an essential first step in the development of suicide prevention programs focused on reducing the stigma of suicide so that they can be adapted to the specific circumstances of each population.

## Author Contributions

**Maitena Pierantonelli**: conceptualization, investigation, writing–original draft, writing–review and editing, data curation, formal analysis, visualization, methodology. **Adriana Mira**: conceptualization, investigation, writing–original draft, writing–review and editing, project administration, supervision, visualization. **Ángel Zamora**: writing–original draft, investigation, conceptualization, formal analysis, data curation, visualization, methodology. **Lorena Desdentado**: data curation, formal analysis, validation, visualization, writing–review and editing, writing–original draft, software, methodology. **Rebeca Diego‐Pedro**: conceptualization, investigation, project administration, resources. **Edgar González‐Hernández**: conceptualization, investigation, project administration, resources. **Juana Bretón‐López**: conceptualization. **Azucena García‐Palacios**: conceptualization. **Rosa M. Baños**: conceptualization, investigation, writing–review and editing, supervision.

### Peer Review

The peer review history for this article is available at https://publons.com/publon/10.1002/brb3.70125.

## Data Availability

The data that support the findings of this study are openly available in Open Science Framework (OSF) at https://osf.io/ketrn/?view_only=f77b403809524222b6dac3fb6e4d4f5c.
